# Abaloparatide exhibits greater osteoanabolic response and higher cAMP stimulation and *β*‐arrestin recruitment than teriparatide

**DOI:** 10.14814/phy2.14225

**Published:** 2019-09-29

**Authors:** Karim Sahbani, Christopher P. Cardozo, William A. Bauman, Hesham A. Tawfeek

**Affiliations:** ^1^ National Center for the Medical Consequences of Spinal Cord Injury James J. Peters Veterans Affairs Medical Center Bronx New York; ^2^ Department of Medicine The Icahn School of Medicine at Mount Sinai New York New York; ^3^ Department of Rehabilitation Medicine The Icahn School of Medicine at Mount Sinai New York New York; ^4^ Department of Pharmacologic Science The Icahn School of Medicine at Mount Sinai New York New York

**Keywords:** Abaloparatide, osteoporosis, PTH, signaling, teriparatide

## Abstract

Teriparatide and abaloparatide are parathyroid hormone receptor 1 (PTHR1) analogs with unexplained differential efficacy for the treatment of osteoporosis. Therefore, we compared the effects of abaloparatide and teriparatide on bone structure, turnover, and levels of receptor activator of nuclear factor‐kappa B ligand (RANKL) and osteoprotegerin (OPG). Wild‐type (WT) female mice were injected daily with vehicle or 20–80 *µ*g/kg/day of teriparatide or abaloparatide for 30 days. Femurs and spines were examined by microcomputed tomography scanning and serum levels of bone turnover markers, RANKL, and OPG, were measured by ELISA. Both analogs similarly increased the distal femoral fractional trabecular bone volume, connectivity, and number, and reduced the structure model index (SMI) at 20–80 *µ*g/kg/day doses. However, only abaloparatide exhibited a significant increase (13%) in trabecular thickness at 20 *µ*g/kg/day dose. Femoral cortical evaluation showed that abaloparatide caused a greater dose‐dependent increase in cortical thickness than teriparatide. Both teriparatide and abaloparatide increased lumbar 5 vertebral trabecular connectivity but had no or modest effect on other indices. Biochemical analysis demonstrated that abaloparatide promoted greater elevation of procollagen type 1 intact N‐terminal propeptide, a bone formation marker, and tartrate‐resistant acid phosphatase 5b levels, a bone resorption marker, and lowered the RANKL/OPG ratio. Furthermore, PTHR1 signaling was compared in cells treated with 0–100 nmol/L analog. Interestingly, abaloparatide had a markedly lower EC50 for cAMP formation (2.3‐fold) and *β*‐arrestin recruitment (1.6‐fold) than teriparatide. Therefore, abaloparatide‐improved efficacy can be attributed to enhanced bone formation and cortical structure, reduced RANKL/OPG ratio, and amplified Gs‐cAMP and *β*‐arrestin signaling.

## Introduction

Bone is continuously remodeled to preserve skeletal integrity and maintain calcium (Ca^2+^) homeostasis. The process of bone remodeling is highly coordinated and is initiated by osteoclast, multinucleated cells of hematopoietic origin, responsible for bone resorption. Release of local factors at the remodeling site stimulates the recruitment and differentiation of osteoblast lineage cells, cells of mesenchymal origin, to synthesize new bone matrix with subsequent mineralization (Martin and Sims 2005; Henriksen et al. 2009; Martin et al. 2009; Sims and Vrahnas 2014). Predominance of bone resorption over bone formation in old age results in a marked decline in bone mineral density (BMD) and biomechanical properties causing senile osteoporosis. Therefore, antiresorptive and bone anabolic agents that inhibit osteoclast function and stimulate osteoblast maturation, respectively, have been widely used to treat osteoporosis.

Parathyroid hormone (PTH) and PTH‐related peptide (PTHrP) are two important regulators of bone remodeling. PTH is a single‐chain 84 amino acid polypeptide hormone secreted by the parathyroid glands in response to hypocalcemia. The primary function of the endogenous PTH is to act directly on the kidney and bone, and indirectly on the intestine by the action of vitamin D to maintain Ca^2+^ and inorganic phosphate (Pi) homeostasis. On the other hand, PTHrP, as was initially discovered, is only detectible in the circulation under certain types of malignancy that secrete high levels of PTHrP contributing to bone breakdown and paraneoplastic hypercalcemia (Honda et al. 1988; Kukreja et al. 1988; Nagata et al. 1989; Gaich and Burtis 1990; Tsuchihashi et al. 1990; Grill et al. 1991; Burtis et al. 1992). Thus, under normal conditions, PTHrP is produced only locally by a variety of normal fetal and adult tissues and acts in an autocrine and paracrine fashion. The locally secreted PTHrP acts to promote placental Ca^2+^ transport from the mother to the fetus, and to regulate chondrocyte differentiation and development of the growth plate, postnatal bone remodeling, vascular smooth muscle function, mammary gland development, tooth eruption, beta‐cell proliferation, and keratinocyte differentiation (Noda et al. 1994; Lee et al. 1996; Vortkamp et al. 1996; Chung et al. 1998; Foley et al. 1998; Philbrick et al. 1998; Wysolmerski et al. 1998; Lanske et al. 1999; Foley et al. 2001; Cebrian et al. 2002; Miao et al. 2005; Hens et al. 2007; Meziani et al. 2008; Hirai et al. 2011; Williams et al. 2011; Raison et al. 2013; Boras‐Granic et al. 2014; Mozar et al. 2016). PTHrP exists in three isoforms of 139, 141, or 173 amino acids due to alternative promoter activity and mRNA splicing.

Both PTH and PTHrP act by activating a common receptor known as the PTH/PTHrP receptor or PTH receptor 1 (PTHR1), a G protein‐coupled receptor that activates multiple downstream pathways and effectors (Abou‐Samra et al. 1992; Sneddon et al. 2000; Tawfeek et al. 2001; Tawfeek et al. 2002; Ahmed et al. 2003; Sneddon et al. 2004; Tawfeek and Abou‐Samra 2004; Singh et al. 2005; Gesty‐Palmer et al. 2006; Rey et al. 2006; Sneddon and Friedman 2007; Sneddon et al. 2007; Tawfeek and Abou‐Samra 2008; Garrido et al. 2009; Tawfeek and Abou‐Samra 2012). PTH effects on bone remodeling involve multiple molecules and complex interaction between osteoblast progenitors, osteoblasts, osteocytes, osteoclasts, and T cells (Locklin et al. 2003; Lotinun et al. 2004; Bellido et al. 2005; Bouxsein et al. 2005; Bellido 2006; Jilka et al. 2009; Terauchi et al. 2009; Bedi et al. 2010; Tawfeek et al. 2010; Jilka et al. 2010; Bohinc and Gesty‐Palmer 2011; Robling et al. 2011; Bedi et al. 2012; Hanyu et al. 2012). However, osteoblast lineage cells and their PTHR1 remain central for PTH and PTHrP effects on bone formation and resorption. The latter is due to PTH or PTHrP stimulation of osteoclastogenic factors produced by the osteoblast lineage cells.

The effects of PTH and PTHrP on bone remodeling are of high clinical interest. This interest emerges from the fact that both PTH and PTHrP cause bone loss when continuously secreted during hyperparathyroidism and hypercalcemia of malignancy, respectively. In contrast, the amino‐terminal fragments of these two proteins (PTH1‐34, PTHrP1‐34, and PTHrP1‐36) offer a treatment for osteoporosis if administered intermittently. Specifically, repeated transient elevation of PTH or PTHrP (Plotkin et al. 1998; Stewart et al. 2000; Horwitz et al. 2003; Horwitz et al. 2010; Horwitz et al. 2013) achieved by daily administration to animals or humans stimulates predominantly bone formation and increases primarily trabecular bone mass.

For these reasons, a daily regimen of PTH (1–34) and PTHrP (1–34) derivative peptide analogs, teriparatide and abaloparatide, respectively, have attained Food and Drug Administration (FDA) approval for the treatment of severe postmenopausal osteoporosis (Neer et al. 2001; Leder et al. 2015; Cosman et al. 2017; Bilezikian et al. 2018). Abaloparatide shares 41% homology to teriparatide and 71% to the parent PTHrP1‐34 but with modifications of five amino acids between residues 22 and 34. Abaloparatide demonstrated more favorable effects on the skeleton in animals and humans with more potent anabolic activity and limited bone resorptive and calcemic response when compared to teriparatide (Leder et al. 2015; Bahar et al. 2016; Miller et al. 2016; Cosman et al. 2017; Varela et al. 2017; Chandler et al. 2018; Doyle et al. 2018). The mechanisms underlying the superior actions of abaloparatide over teriparatide remain elusive. In this study, we used an in vivo and in vitro approach to evaluate, in parallel, the effects of teriparatide and abaloparatide on bone structure and microarchitecture, bone turnover, and PTHR1 signaling.

## Materials and Methods

### Reagents

Abaloparatide and teriparatide were chemically synthesized (LifeTein, NJ) and purified by reverse‐phase chromatography (>95% pure). The molecular mass of the peptide was verified by mass spectrometry, and the net peptide content and composition were confirmed by amino acid analysis. It is not possible to use the commercially available teriparatide (Forteo, Lilly) and abaloparatide (Tymlos, Radius) in animals due to the low concentration formulation for human use and the prohibitively high cost of the commercial products for human use. Tissue culture media were purchased from GE Healthcare Life Sciences HyClone Laboratories (Logan, Utah). Fetal bovine serum (FBS) (Cat number 900–108, lot number A47D) was from Geminibio‐Products (West Sacramento, CA). Penicillin‐streptomycin (PS, Cat number MT‐30‐001‐CI), Trypsin (0.05%, Cat number 25‐300‐054), and phosphate‐buffered saline (PBS, Cat number 10‐010‐072) were from Lifescience Technologies (Grand Island, NY). 3‐isobutyl‐1‐methylxanthine (IBMX) (Cat number I5879) was from Sigma‐Aldrich (St. Louis, MO). Tissue culture plates and other supplies were from Corning (Oneonta, NY) and Fisher Scientific (Pittsburgh, PA).

### Animals

All animal procedures were reviewed and approved by the Institutional Animal Care and Use Committee at James J. Peters Veterans Affairs Medical Center. All experiments were conducted on 16‐week‐old wild‐type (WT) female C57BL/6J mice (Stock number 664) from Jackson Laboratories (Kingston, NY). Vehicle (0.9% NaCl/10 mmol/L acetic acid) or 20–80 *µ*g/kg/day teriparatide or abaloparatide was injected subcutaneously (SC) daily (except Sunday) and continued for 30 days. No peptide injection was performed on the day of animal sacrifice.

### Cell culture

MC3T3‐E1 subclone 4 osteoblast cell line (Cat number ATCC® CRL‐2593) was purchased from American Type Culture Collection (ATCC) (Manassas, VA). All cell cultures were performed in alpha‐modified Eagle's medium (alpha‐MEM) (Cat number SH30265.02) from GE Healthcare Life Science Hyclone Laboratories (Logan, Utah) supplemented with 10% FBS and 1% PS. Cells were incubated at 37°C using a humidified atmosphere containing 95% air and 5% CO_2_. Cell passage was performed every 7 days (1 million cells/75 cm flask) and cells were not used beyond passage 8.

### Micro‐CT measurements of trabecular and cortical bone structure

Animals were euthanized using isoflurane inhalation (to effect) anesthesia followed by exsanguination by cardiac puncture and cervical dislocation. Harvested femurs were fixed in 10% neutral‐buffered formalin for 4 h at RT and 24 h at 4°C, rinsed extensively in tap water, and preserved in 70% ethanol at 4°C until analysis. Bones were scanned using a microcomputed tomographic (micro‐CT) instrument (VivaCT‐40, Scanco Medical AG, Bassersdorf, Switzerland). The VivaCT‐40 is calibrated weekly using a phantom provided by Scanco. Trabecular bone volume fraction and microarchitecture in the lumbar 5 vertebra (L5) and distal femoral region and cross‐sectional geometry at the femoral mid‐shaft were assessed. Scanning for the distal femoral trabecular region was performed in the secondary spongiosa starting approximately at 0.6 mm proximal to the growth plate and extending proximally 1.5 mm. Scans for the cortical region were performed at the midpoint of each femur and an isotropic pixel size of 10.5 *µ*m and slice thickness of 10.5 *µ*m were used to calculate the average total cross‐sectional area (mm^2^), bone area (mm^2^), and marrow area (mm^2^). The bones were scanned at high resolution with energy level of 70 kVp, and intensity of 114 *μ*A and 8 W at 10.5 *μ*m voxel size, and integration time 200 msec. Trabecular 150 consecutive slices at 10.5‐*µ*m interval were evaluated with a threshold of 165 density units. Cortical 50 consecutive slices were evaluated with a threshold of 700 density units. Standard nomenclature and guidelines for assessment of bone microstructure were followed, as recommended by the American Society for Bone and Mineral Research (Bouxsein et al. 2010). The main bone parameters are TV (total volume, mm^3^), BV (bone volume, mm^3^), BV/TV (relative volume of calcified tissue in the selected volume of interest (VOI)), Tb. Th (thickness of the trabecular structure, mm), Tb. N (number of trabeculae/mm), Tb. Sp (trabecular separation or spacing; a measurement of the thickness of the spaces between the trabeculae, mm), Conn. D (connectivity density; 3‐D connectivity index; a measure of the degree to which a structure is multiply connected, normed by TV, 1/mm^3^), TRI‐SMI (three‐dimensional structure model index (SMI), related to the architecture of the structure and ranges between 0 and 3, SMI toward 0 signifies that the structure is mainly concave plates, whereas a value of 3 means only cylindrical rods), Ct. Th (cortical thickness, mm), Ct. Po (Cortical porosity in a given cortical region, the volume of pores (Po.V, mm^3^)/total volume of cortical bone compartment (Ct.V, mm^3^).

### Measurement of serum markers of bone turnover

Blood was drawn from isoflurane‐anesthetized animals using cardiac puncture as a terminal procedure. Blood was collected in BD Microtainer™ Capillary Blood Collector and BD Microgard™ Closure from Fisher Scientific (Fisher Cat number 02‐675‐185 and BD manufacturer Cat number BD 365967). Serum was separated after spinning in a microcentrifuge for 10 min at 4°C and then frozen at −80°C. Serum markers of bone turnover were measured, as described previously (Gao et al. 2008; Terauchi et al. 2009; Tawfeek et al. 2010) and by following the manufacturer's instructions. Serum procollagen type 1 intact N‐terminal propeptide (P1NP), a specific marker of bone formation, collagen type I C‐telopeptide or carboxy‐terminal collagen crosslinks (CTX), a marker of bone resorption, and tartrate‐resistant acid phosphatase (TRAcP‐5b), a specific serum marker of osteoclastic activity, were measured using rodent‐specific ELISA assay kits (Cat numbers AC33F1, AC‐06F1, SB‐TR103, respectively) from Immunodiagnostic Systems (Scottsdale, AZ). Serum levels of receptor activator of nuclear factor‐kappa B ligand (RANKL) and osteoprotegerin (OPG) were measured using Mouse TRANCE/RANK L/TNFSF11 (Cat number MTR00) and mouse osteoprotegerin/TNFRSF11B (Cat number MOP00) quantikine ELISA kits, respectively, from R&D Systems, Inc. (Minneapolis, MN).

### Measurement of serum Ca^2+^ and Pi

Blood was collected and serum was separated as described above. Serum Ca^2+^ and Pi concentrations were measured using colorimetric Ca^2+^ and Pi assay kits (Cat numbers K380 and K410, respectively) as indicated by the manufacturer (BioVision, Milpitas, CA).

### Measurement of intracellular cAMP generation

MC3T3‐E1 cells were seeded at 40,000 cells/well of a 24‐well plate containing 500‐*µ*L alpha‐MEM supplemented with 10% FBS and 1% PS. After culture for 1 week, the medium was removed and replaced with 250 *µ*L of stimulation medium (alpha‐MEM containing 0.05% FBS, 0.1% BSA, 5 mmol/L hepes buffer, and 0.5 mmol/L IBMX) for 15 min. IBMX is a phosphodiesterase inhibitor that prevents degradation of the generated cAMP. Vehicle, abaloparatide, and teriparatide were then added in 250 *µ*L stimulation medium to achieve final concentrations of 0, 0.01, 0.1, 1, 10, and 100 nmol/L/well. Incubation continued for 40 min at 37°C before the medium was removed and the plates were snap frozen in liquid N3 and stored at −80°C. For extraction of intracellular cAMP, 100 mmol/L Hcl was added and cells were incubated at room temperature for 1 h. Intracellular cAMP was assayed using a cAMP competitive ELISA kit (Cat number EMSCAMPL) from Thermo Fisher Scientific (Waltham, MA) and following the manufacturer protocol and instructions.

### PathHunter^®^ eXpress PTHR1 CHO‐K1 *β*‐arrestin GPCR assay

To assess the effects of abaloparatide and teriparatide stimulation of PTHR1 on *β*‐arrestin recruitment to the cell membrane, a PathHunter eXpress PTHR1 Chinese Hamster Ovary‐K1 (CHO‐K1) *β*‐arrestin GPCR Assay from Eurofins DiscoverX (Fremont, CA) (Cat Number 93‐0315E2CP0S) was used. The assay takes advantage of Enzyme Fragment Complementation technology. The PTHR1 is fused in frame with a small enzyme donor fragment ProLink™ (PK) and co‐expressed in CHO‐K1 cells stably expressing a fusion protein of *β*‐arrestin and the larger, N‐terminal deletion mutant of *β*‐galactosidase (called enzyme acceptor or EA). Activation of the PTHR1 stimulates binding of *β*‐arrestin to the PK‐tagged GPCR and forces complementation of the two enzyme fragments, resulting in the formation of an active *β*‐galactosidase enzyme. An increase in enzyme activity is then measured using chemiluminescent PathHunter Detection Reagents. Cell seeding, incubation, and detection were performed as instructed by the manufacturer. Briefly, cells were seeded in a clear bottom white 96‐well plate and incubated for 48 h at 37°C CO2 incubator. Cells were treated with vehicle, teriparatide, or abaloparatide for 60 min at 37°C in a CO2 incubator. At the end of the incubation, *β*‐gal enzyme substrate was added for 60 min at room temperature in the dark. Light generation (Relative Light Units, RLU), an indication of *β*‐gal enzyme fragment complementation and *β*‐Arrestin/ PTHR1 interaction, was measured using BMG Labtech PHERAstar FS luminescence plate reader.

### PathHunter® eXpress PTHR1 activated GPCR internalization assay

To determine PTHR1 internalization, we used PathHunter eXpress PTHR1 U2OS Activated GPCR Internalization Assay (Cat number 93‐0770E3CP0S) from Eurofins DiscoverX (Fremont, CA). PathHunter^®^ PTHR1 Activated GPCR Internalization U2OS cell lines are engineered to co‐express an untagged PTHR1, an EA‐tagged *β*‐arrestin, and a PK tag localized to the endosomes. Activation of the untagged PTHR1 induces *β*‐arrestin recruitment, followed by internalization of the GPCR‐*β*‐arrestin‐EA complex in PK‐tagged endosomes. Similar to the *β*‐arrestin assay format, this internalization forces complementation of the two *β*‐gal enzyme fragments, forming functional enzyme that hydrolyzes substrate to generate a chemiluminescent signal. U2OS osteoblastic cell line seeding, incubation, and detection were performed as instructed by the manufacturer. Cells were treated with vehicle, teriparatide, or abaloparatide for 60 min at 37°C in a CO2 incubator. At the end of the incubation, *β*‐gal enzyme substrate was added for 60 min at room temperature in the dark. Light generation (RLU), an indication of *β*‐gal enzyme fragment complementation and *β*‐arrestin/endosome/PTHR1 formation, was measured using BMG Labtech PHERAstar FS luminescence plate reader.

### Statistical analysis

As per experimental protocol, animals were randomized between vehicle, teriparatide, and abaloparatide groups and the analyses were performed blindly regarding the control and test groups using unique animal identification codes. For in vivo studies, five mice/group were used. For in vitro cell biological and biochemical studies, each experiment was repeated three to four times and in each experiment, every experimental condition was performed in duplicate or triplicate. Statistical analyses were performed using GraphPad Prism and Microsoft excel. EC50 represents the molar concentration at which a substance exerts half of its maximal response and is an indication of drug potency. EC50 was calculated using nonlinear regression analyses of normalized cAMP dose–response curves of abaloparatide and teriparatide. The results are presented as the means ± SD. Unpaired Student *t*‐test was used for comparison of EC50 between two teriparatide and abaloparatide groups. For carrying out multiple group comparisons (all in vivo experiments), one‐way analysis of variance (ANOVA) was performed. When ANOVA indicated unequal means, Tukey's honestly significant difference (HSD) posthoc test was performed to determine which means were different to the level of significance. Statistical significance was considered when *P* value was less than 0.05 (*P* < 0.05).

## Results

### Abaloparatide and teriparatide similarly improve trabecular bone architecture

**Figure 1:**
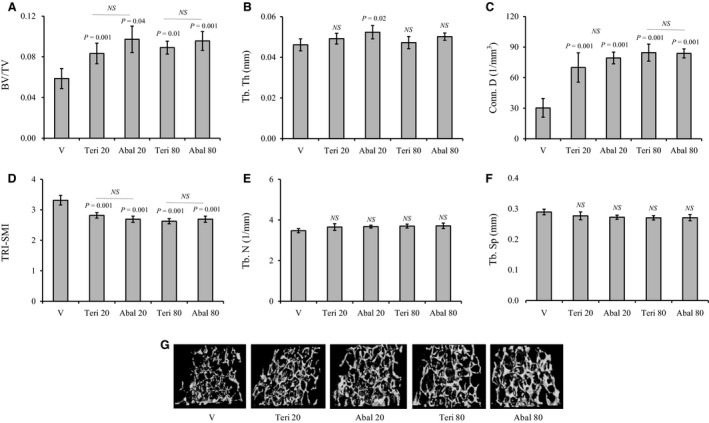
Effects of abaloparatide and teriparatide administration on femoral trabecular bone architecture: Female C57BL/6J WT mice were SC injected daily, except Sunday, with vehicle or 20–80 *µ*g/kg/day teriparatide or abaloparatide for 30 days. Femurs were isolated, fixed, and distal femur metaphysis regions were analyzed by micro‐CT. (A) BV/TV is trabecular bone volume/total volume. (B) Tb. Th is trabecular thickness. (C) Conn. D is trabecular connectivity density. (D) SMI is trabecular three‐dimensional structure model index. (E) Tb. N is trabecular number. (F) Tb. Sp is trabecular spacing or separation. (G) Images: Examples of distal femur micro‐CT scans. The statistical designation directly listed on the top of each bar indicates the significance level in comparison to vehicle. Statistical significance levels between other experimental groups are shown using the drawn lines. The data in all graph are expressed as the means ± SD. *N* = 5 mice/group. NS is not significant. V is vehicle, Teri 20 is teriparatide 20 *µ*g/kg/day, Abal 20 is abaloparatide 20 *µ*g/kg/day, Teri 80 is teriparatide 80 *µ*g/kg/day, and Abal 80 is abaloparatide 80 *µ*g/kg/day.

To assess the effects of intermittent abaloparatide and teriparatide administration on trabecular bone structure, WT female C57BL/6J mice were injected daily (except Sunday) SC with vehicle or 20–80 *µ*g/kg/day of teriparatide or abaloparatide for 30 days. Mice were then sacrificed and the distal metaphyseal regions of the isolated femurs and L5 vertebrae were analyzed by micro*‐*CT. Abaloparatide and teriparatide administration at 20 or 80 *µ*g/kg/day similarly increased fractional trabecular bone volume (BV/TV) and trabecular connectivity (Conn. D), and reduced the three‐dimensional SMI (Fig. 1A, C, and D). There was no detectible changes in trabecular number (Tb. N) or spacing (Tb. Sp) among all treatment groups (Fig. 1E and F). However, Tb. Th was the only trabecular index that was marginally responsive to abaloparatide treatment at 20 (113%) or 80 *µ*g/kg/day (108%) when comparing either dose group to vehicle‐treated controls (Fig. 1B). Qualitative changes in femoral trabecular bone structure are shown in micro‐CT images of the distal femoral regions (Fig. 1G).

**Figure 2:**
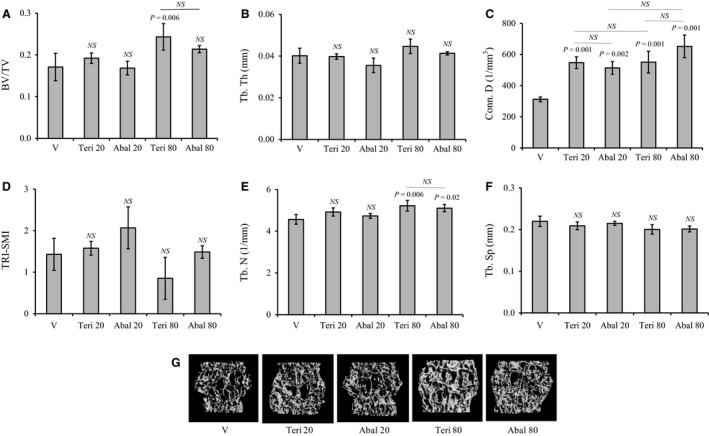
Effects of abaloparatide and teriparatide administration on vertebral trabecular bone architecture: Female C57BL/6J WT mice were SC injected daily, except Sunday, with vehicle or 20–80 *µ*g/kg/day teriparatide or abaloparatide for 30 days. L5 vertebrae were isolated, fixed, and analyzed by micro‐CT. (A) BV/TV is trabecular bone volume/total volume. (B) Tb. Th is trabecular thickness. (C) Conn. D is trabecular connectivity density. (D) SMI is trabecular three‐dimensional structure model index. (E) Tb. N is trabecular number. (F) Tb. Sp is trabecular spacing or separation.(G) Images: Examples of L5 micro‐CT scans. The statistical designation directly listed on the top of each bar indicates the significance level in comparison to vehicle. Statistical significance levels between other experimental groups are shown using the drawn lines. The data in all graph are expressed as the means ± SD. *N* = 5 mice/group. NS is not significant. V is vehicle, Teri 20 is teriparatide 20 *µ*g/kg/day, Abal 20 is abaloparatide 20 *µ*g/kg/day, Teri 80 is teriparatide 80 *µ*g/kg/day, and Abal 80 is abaloparatide 80 *µ*g/kg/day.

Overall, the effects of teriparatide and abaloparatide on L5 structure and microarchitecture were modest compared to the femur (Fig. 2A–G). While, only animals receiving 80 *µ*g/kg/day teriparatide had a significant (*P* = 0.006) elevation (42%) in vertebral BV/TV when compared to vehicle, this effect was not significant when compared to 80 *µ*g/kg/day abaloparatide counterparts (Fig. 2A). Furthermore, Conn. D was greatly augmented by 64–100% in response to 20 or 80 *µ*g/kg/day teriparatide or abaloparatide administration (Fig. 2C). Tb. N was equally increased only in 80 *µ*g/kg/day teriparatide‐ and abaloparatide‐treated animals (Fig. 2E). Conversely, L5 Tb. Th, TRI‐SMI, and Tb. Sp were not altered in any animal group irrespective of the treatment (Fig. 2B, D, and F). Micro‐CT images show the qualitative changes in bone structure of L5 vertebrae (Fig. 2G).

### Abaloparatide treatment causes larger increase in cortical bone thickness than teriparatide

**Figure 3:**
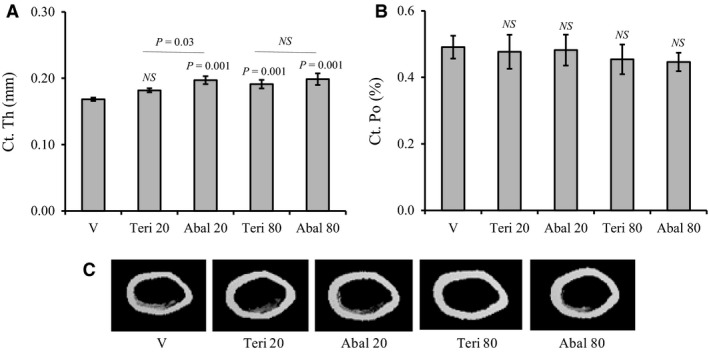
Changes in cortical bone structure in response to abaloparatide and teriparatide administration: Female C57BL/6J WT mice were injected SC daily, except Sunday, with vehicle or 20–80 *µ*g/kg/day teriparatide or abaloparatide for 30 days. Femurs were isolated, fixed, and mid‐shaft regions were analyzed by micro‐CT. (A) Ct. Th is cortical thickness. (B) Ct. Po is cortical porosity. (C) Images: Examples of femur mid‐shaft micro‐CT scans. The statistical designation directly listed on the top of each bar indicates the significance level in comparison to vehicle. Statistical significance levels between other experimental groups are shown using the drawn lines. The data in all graph are expressed as the means ± SD. *N* = 5 mice/group. NS is not significant. V is vehicle, Teri 20 is teriparatide 20 *µ*g/kg/day, Abal 20 is abaloparatide 20 *µ*g/kg/day, Teri 80 is teriparatide 80 *µ*g/kg/day, and Abal 80 is abaloparatide 80 *µ*g/kg/day.

To determine the effects of abaloparatide and teriparatide treatment on cortical bone structure, mid‐shaft regions of isolated femurs were analyzed by micro*‐*CT. Abaloparatide administration efficiently expanded cortical thickness (Ct. Th) at both doses of 20 and 80 *µ*g/kg/day by 17% and 18%, respectively (Fig. 3A). In contrast, teriparatide at 20 *µ*g/kg/day failed to achieve a significant increase in Ct. Th (Fig. 3A). The effects of teriparatide and abaloparatide on Ct. Th at 80 *µ*g/kg/day dose were not statistically different (Fig. 3A). No effects were observed on cortical porosity (Ct. Po) for either abaloparatide or teriparatide treatment at 20 or 80 *µ*g/kg/day dose regimen (Fig. 3B). Qualitative changes in cortical bone structure are shown in micro‐CT images of the femoral mid‐shaft regions (Fig. 3C).

### Abaloparatide produces higher bone formation and lower RANKL/OPG ratio than teriparatide

**Figure 4:**
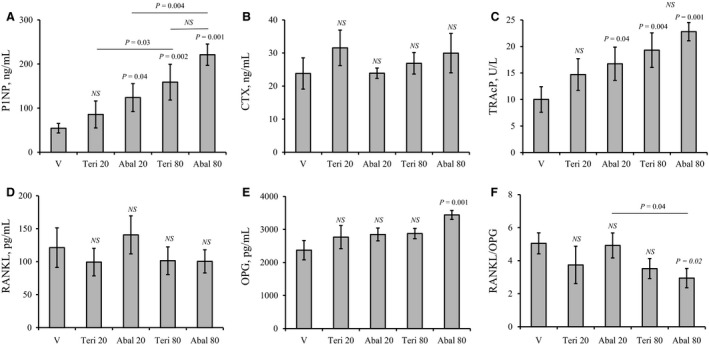
Abaloparatide and teriparatide stimulation of serum markers of bone turnover, RANKL and OPG: Female C57BL/6 WT mice were injected SC daily, except Sunday, with vehicle or 20–80 *µ*g/kg/day teriparatide or abaloparatide for 30 days. Sera were collected and serum levels of bone formation and resorption markers, RANKL, and OPG were determined using specific ELISA kits. (A) Serum levels of P1NP. (B) Serum levels of CTX. (C) Serum levels of TRAcP‐5b. (D) Serum levels of RANKL. (E) Serum levels of OPG. (F) RANKL/OPG ratio. The statistical designation directly listed on the top of each bar indicates the significance level in comparison to vehicle. Statistical significance levels between other experimental groups are shown using the drawn lines. The data in all graph are expressed as the means ± SD. *N* = 5 mice/group. NS is not significant. V is vehicle, Teri 20 is teriparatide 20 *µ*g/kg/day, Abal 20 is abaloparatide 20 *µ*g/kg/day, Teri 80 is teriparatide 80 *µ*g/kg/day, and Abal 80 is abaloparatide 80 *µ*g/kg/day.

To determine the effects of teriparatide and abaloparatide administration on the levels of serum markers of bone turnover, OPG, and RANKL, specific ELISA kits were used. Analysis of sera collected from WT female mice after 30 days of vehicle, teriparatide, or abaloparatide treatment showed that only abaloparatide achieved a significant dose‐dependent increase in P1NP, a specific bone formation marker (Fig. 4A). Abaloparatide increased P1NP levels to 227% and 407% at 20 and 80 *µ*g/kg/day, respectively. On the other hand, teriparatide stimulation of P1NP was not statistically significant at 20 *µ*g/kg/day dose but a 291% elevation of P1NP was achieved when teriparatide was used at 80 *µ*g/kg/day dose (*P* = 0.002) (Fig. 4A). Neither abaloparatide nor teriparatide was able at any dose to alter serum levels of CTX, a bone resorption marker (Fig. 4B). However, measurement of serum levels of TRAcP‐5b, a specific marker of osteoclast activity, demonstrated that abaloparatide treatment efficiently increased TRAcP‐5b levels at both 20 (167%) and 80 *µ*g/kg/day (227%) doses (Fig. 4C). Teriparatide‐treated animals, however, had increased TRAcP‐5b levels (193%) only at 80 *µ*g/kg/day dose (Fig. 4C). Of note, there was no statistical difference between abaloparatide and teriparatide TRAcP‐5b response at 80 *µ*g/kg/day dose or between abaloparatide responses at 20 and 80 *µ*g/kg/day doses.

Assessment of serum levels of RANKL revealed that neither abaloparatide nor teriparatide had any significant effects on RANKL levels (Fig. 4D). On the other hand, only abaloparatide at 80 *µ*g/kg/day increased OPG levels by 44% when compared to vehicle (Fig. 4E). Consistently, calculation of relative serum levels of RANKL and OPG further demonstrated that only 80 *µ*g/kg/day abaloparatide treatment intervention was able to lower RANKL/OPG ratio by 42% (Fig. 4F). A reduction of RANKL/OPG ratio by 80 *µ*g/kg/day of teriparatide administration was not statistically significant (*P* = 0.2).

### No significant differences between teriparatide and abaloparatide treatment on mineral homeostasis

**Figure 5:**
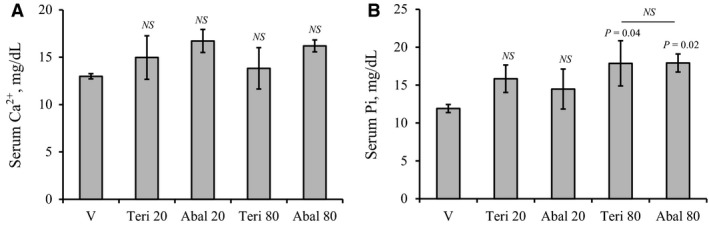
Effects of daily abaloparatide and teriparatide on serum Ca^2+^ and Pi: Female C57BL/6 WT mice were injected SC daily, except Sunday, with vehicle or 20–80 *µ*g/kg/day teriparatide or abaloparatide for 30 days. Sera were collected at sacrifice (24 h after last injection) and levels of Ca^2+^ and Pi were measured using specific colorimetric assays. (A) Serum Ca^2+^ levels. (B) Serum Pi levels. The statistical designation directly listed on the top of each bar indicates the significance level in comparison to vehicle. Statistical significance levels between other experimental groups are shown using the drawn lines. The data in all graph are expressed as the means ± SD. *N* = 5 mice/group. NS is not significant. V is vehicle, Teri 20 is teriparatide 20 *µ*g/kg/day, Abal 20 is abaloparatide 20 *µ*g/kg/day, Teri 80 is teriparatide 80 *µ*g/kg/day, and Abal 80 is abaloparatide 80 *µ*g/kg/day.

Ca^2+^ and Pi levels were measured, as described in the methods section, in the serum samples collected at the end of the 30‐day injection. No significant differences in serum Ca^2+^ levels were detected when all animal groups treated with vehicle, abaloparatide, and teriparatide were compared (Fig. 5A). However, a statistically significant (*P* = 0.006) 24% increase in serum Ca^2+^ of 20 and 80 *µ*g/kg/day abaloparatide groups was detected when compared to only vehicle controls (Fig. 5A). The effects on serum Pi were observed only in animals receiving 80 *µ*g/kg/day teriparatide or abaloparatide (Fig. 5B). Administration of 80 *µ*g/kg/day of either teriparatide or abaloparatide resulted in a considerable 50% elevation in Pi levels when compared to vehicle‐treated animals (Fig. 5B).

### Enhanced Gs/cAMP signaling and *β*‐arrestin recruitment in abaloparatide‐treated cells

**Figure 6:**
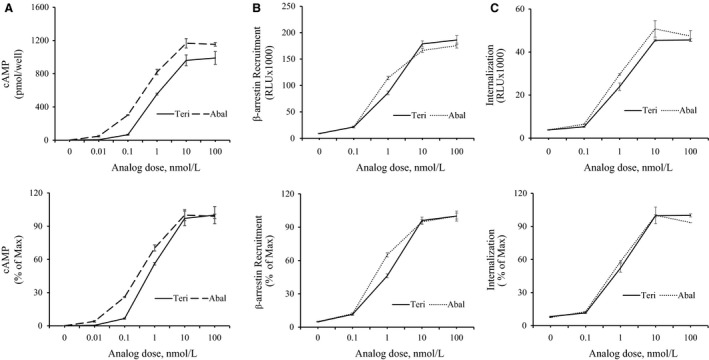
Activation of PTHR1 signaling by abaloparatide and teriparatide treatment: (A) Activation of intracellular cAMP production: MC3T3‐E1 cells were challenged with 0–100 nmol/L of abaloparatide or teriparatide for 40 min at 37°C in the presence of IBMX. The medium was disposed and cells were snap frozen on liquid N3 before storage at 80°C. Intracellular cAMP was extracted and measured using ELISA assay as described by the manufacturer protocol. Each dose treatment was performed in duplicate and the experiment was repeated three times. The EC50 was calculated using results from three independent experiments. Results are the means ± SD. Upper panel: Absolute values are expressed as pmol/well. Lower panel: Values are expressed as % stimulation/maximal response. (B) Activation of *β*‐arrestin recruitment: PathHunter eXpress PTHR1 CHO‐K1 *β*‐arrestin GPCR assay was described under the material and methods section. CHO‐K1 cells were treated with 0–100 nmol/L of teriparatide or abaloparatide for 60 min at 37°C. Teriparatide and abaloparatide stimulations of *β*‐arrestin recruitment were determined by measuring light generation after adding *β*‐gal enzyme substrate. The EC50 was calculated using results from three independent experiments. Results are the means ± SD. Upper panel: Absolute values are expressed as relative light units (RLU). Lower panel: Values are expressed as % stimulation/maximal response. (C) Stimulation of PTHR1 internalization: PathHunter eXpress PTHR1 U2OS activated GPCR internalization assay was described under the material and methods section. U2OS cells were treated with 0–100 nmol/L of teriparatide or abaloparatide for 60 min at 37°C. PTHR1 internalization was determined by measuring light generation after adding *β*‐gal enzyme substrate. The EC50 was calculated using results from three independent experiments. Results are the means ± SD. Upper panel: Absolute values are expressed as RLU. Lower panel: Values are expressed as % stimulation/maximal response.

MC3T3‐E1 osteoblast cells were treated with vehicle or 0.01–100 nmol/L of abaloparatide or teriparatide for 40 min at 37°C in the presence of 0.5 mmol/L IBMX. The results revealed that exposure of cells to abaloparatide or teriparatide caused a robust elevation of intracellular cAMP levels (Fig. 6A, upper panel). Increasing doses of abaloparatide caused a shift to the left of the cAMP dose–response curve compared to teriparatide‐treated cell response (Fig. 6A, lower panel). As a result, abaloparatide treatment resulted in a 2.3‐fold decrease in EC50 value for cAMP formation compared to teriparatide (EC50 = 0.3 ± 0.03 nmol/L and 0.7 ± 0.2 nmol/L, respectively, *p = 0.02*). Maximum cAMP stimulation, however, was not significantly different between the two analogs (Fig. 6A, upper panel).

*β*‐arrestin is an important cytosolic protein that interacts with PTHR1 and mediates PTHR1 internalization, desensitization, and signaling to ERK1/2 MAPK pathway (Ferrari et al. 1999; Gesty‐Palmer et al. 2006; Rey et al. 2006; Sneddon et al. 2007; Tawfeek and Abou‐Samra, 2012). To assess the effects of abaloparatide and teriparatide stimulation of PTHR1 on *β*‐arrestin recruitment, a PathHunter eXpress PTHR1 CHO‐K1 *β*‐arrestin GPCR Assay was used as described under the methods section. A dose‐dependent stimulation of *β*‐arrestin/PTHR1 interaction was demonstrated by abaloparatide and teriparatide analogs (Fig. 6B, upper panel). Compared to teriparatide, abaloparatide treatment caused a shift to the left of the dose–response curve (Fig. 6B, lower panel). Consistently, the calculated the EC50 value for abaloparatide was 1.6‐fold lower than that of teriparatide (EC50 = 0.9 ± 0.2 nmol/L and 1.5 ± 0.3 nmol/l, respectively, *P* = 0.02). The maximal stimulation for both analogs was reached at equimolar dose of a 100 nmol/L.

To measure PTHR1 internalization, PathHunter^®^ PTHR1 Activated GPCR Internalization U2OS Cell Line was used as described under the methods section. Both abaloparatide and teriparatide efficiently induced a dose‐dependent stimulation of PTHR1 internalization with a dose as low as 0.1 nmol/L and reached maximum stimulation at 100 nmol/L concentration (Fig. 6C, upper panel). There was, however, no significant difference between abaloparatide and teriparatide activation of PTHR1 internalization (Fig. 6C, lower panel). The EC50 values for both abaloparatide (EC50 = 0.8 ± 0.4 nmol/L) and teriparatide (EC50 = 1.1 ± 0.4 nmol/L) stimulation of internalization were not statistically different.

## Discussion

In this report, a combination of in vivo and in vitro approaches was used to identify the mechanisms mediating the differential clinical efficacy of the two PTHR1‐related agents, abaloparatide and teriparatide. Using a mouse model, we demonstrate that the skeletal response to abaloparatide treatment was more amplified compared to teriparatide‐treated animals. A daily regimen of abaloparatide administration caused a larger increase in trabecular and cortical bone thickness and higher bone formation than teriparatide. In vitro studies further demonstrated that abaloparatide activation of PTHR1 produces more efficient coupling of PTHR1 to Gs‐cAMP and *β*‐arrestin signaling mechanisms than that for teriparatide.

Human life expectancy has increased, thanks to the advances in medical research and the subsequent improvement in health care. As a result, there is a growing demand for better management of the common illnesses of the increasing elderly population. One of these illnesses, osteoporosis, is a common world‐wide bone disorder and a major public health threat for 44 million Americans, 68% of them being women. Osteoporosis accounts for an estimated 1.5 million fractures in the US every year costing over 14 billion dollars. Furthermore, the National Osteoporosis Foundation estimates that 50% of women and 25% of men will break a bone in their lifetime due to osteoporosis. The development of drugs that efficiently control bone resorption and/or enhance bone formation will lead to prevention of fractures. In this regard, various antiresorptive drugs, such as bisphosphonates, estrogens, calcitonin, and human monoclonal antibodies (e.g., denosumab to RANKL), have become available. Although these antiresorptive agents prevent further bone loss by inhibiting osteoclast function, they cannot completely restore bone structure. The reduction in fracture risk has also not been satisfactory and some drugs have serious side effects. In light of these drawbacks and the search for anabolic option, animal studies demonstrated that a regimen of daily administration of PTH promotes osteoblast cell differentiation and survival leading to enhanced bone formation and improved bone biomechanical properties (Liu and Kalu 1990; Hirano et al. 1999; Jilka et al. 1999; Bellido et al. 2003; Iida‐Klein et al. 2007; Jilka 2007; Jilka et al. 2010; Kim et al. 2012). Subsequent evaluation of patients with osteoporosis further confirmed that PTH increases BMD in the spine and femur of men and women more efficiently than treatment with bisphosphonates, and decreased the risk of vertebral and nonvertebral fractures by more than 50% (Neer et al. 2001). As a result, the amino‐terminal fragment of PTH (PTH1‐34, teriparatide or Forteo) became the first osteoanabolic treatment approved by the US FDA for treatment of patients with primary (Neer et al. 2001) or secondary osteoporosis due to glucocorticoid therapy who have a high risk of fracture (Saag et al. 2007).

Recently, abaloparatide, a novel modified PTHrP1‐34 and PTHR1 agonist analog, has emerged as another attractive candidate with potent anabolic activity and limited bone resorptive and calcemic response when compared to teriparatide (Leder et al. 2015; Bahar et al. 2016; Miller et al. 2016; Cosman et al. 2017; Varela et al. 2017; Chandler et al. 2018; Doyle et al. 2018). Abaloparatide was therefore recently approved by the US FDA for treatment of severe osteoporosis.

One of these studies was an 18‐month randomized, double‐blind placebo‐controlled Abaloparatide Comparator Trial in Vertebral Endpoints (ACTIVE) trial of more than 2000 women with postmenopausal osteoporosis. Abaloparatide was associated with an 86% reduction in vertebral fracture incidence, the primary end point, compared with placebo (Cosman et al. 2017). Additionally, abaloparatide administration resulted in significantly greater reductions in nonvertebral fractures compared with teriparatide administration and minimal activation of bone resorption (Leder et al. 2015; Cosman et al. 2017; Bilezikian et al. 2018). Our findings suggest that the higher reduction in fracture risk by abaloparatide over teriparatide is likely attributed to its improvement in bone microarchitecture, primarily trabecular and cortical bone thickness. The expansion in Tb. Th produced by the 20 but not the 80 *µ*g/kg/day dose of abaloparatide might be ascribed to stimulation of a catabolic bone mechanism(s) by the higher abaloparatide concentration. Furthermore, the more pronounced effects of abaloparatide than teriparatide on the cortical bone compartment could explain the higher reduction in long bone fracture risk achieved by abaloparatide. The more amplified cortical bone effects of abaloparatide also suggest that periosteal postmitotic pre‐osteoblasts are likely more responsive to abaloparatide stimulation. Periosteal postmitotic pre‐osteoblasts have previously been implicated in daily PTH effects on cortical bone formation due to stimulation of progenitor differentiation and/or survival (Jilka et al. 2009). A decrease in the plate/rod ratio (high SMI) and connectivity are believed to be major contributing factors to fragility fracture in various forms of osteoporosis and with advancing age, while an increase in these parameters induced by treatment with bone anabolic and antiresorptive agents is beneficial for bone quality and strength (Ding and Hvid 2000; Benito et al. 2003; Borah et al. 2004; Rupprecht et al. 2006; Benhamou 2007; Liu et al. 2013; Altman et al. 2014; Stein et al. 2014; Sutter et al. 2014; Zhou et al. 2014; Chang et al. 2015). Our results suggest that both abaloparatide and teriparatide similarly and effectively enhance femoral trabecular connectivity and modify femoral bone structure in favor of concave plate‐like structures. In contrast, the effects of teriparatide and abaloparatide on the spine were predominant on the vertebral trabecular connectivity and number; the actions of the two agents on BV/TV, Tb. Th, Tb. Sp, and SMI were either modest or absent. This disparity in the axial (L5) and appendicular (femur) bone responsiveness to both agents suggests that unloading and weight bearing may play a role in modifying the skeletal response to PTHR1 activation. The comparable responsiveness of the spine to teriparatide and abaloparatide treatment is consistent with those findings reported recently in ovariectomized rats showing parallel improvement in lumbar BMD and strength by both agents (Makino et al. 2018) and further support the reduced vertebral fracture risk in humans.

Surprisingly, we did not observe changes in CTX serum levels, a commonly used bone resorption marker, in response to abaloparatide or teriparatide administration. On the other hand, TRAcP‐5b, a specific serum marker of osteoclast activation, was more sensitive to abaloparatide stimulation than teriparatide. The difference in the response of CTX and TRAcP‐5b markers to teriparatide and abaloparatide stimulation could be explained by the report that TRAcP‐5b is a more specific and sensitive osteoclast and resorption marker than CTX (Halleen et al. 2001). Alternatively, another report suggested that CTX is more reflective of osteoclast activity while TRAcP‐5b of osteoclast number (Rissanen et al. 2008). Other factors that regulate bone resorption are RANKL and its decoy receptor OPG; both are important cytokines produced primarily by the osteoblast lineage cells. Binding of RANKL to its receptor (RANK) in the osteoclast progenitor cells stimulates osteoclast formation and activation, and inhibits apoptosis leading to enhanced bone resorption. As such, the balance between the two cytokines expressed as RANKL/OPG ratio controls the process of bone resorption. In this regard, the only evidence we report for a possible weaker osteoclast activation and bone resorption by abaloparatide is the decrease in RANKL/OPG ratio produced by abaloparatide administration. Thus, the bone turnover and the RANKL/OPG findings suggest that abaloparatide retains a fine‐tuned (due to low RANKL/OPG) bone resorptive property but possesses superior bone formation capacity over teriparatide. Indeed, it appears that the ability of abaloparatide to more efficiently couple bone resorption and formation than teriparatide accounts for the observed more enhanced bone gain. However, we acknowledge that a more comprehensive analysis including longer and shorter time course is needed for careful evaluation of the catabolic and anabolic activity of both abaloparatide and teriparatide. Moreover, whether the catabolic effects of abaloparatide would be modified by the prior status of bone resorption, such as in estrogen‐deficient (ovariectomized) mice, remains to be elucidated.

Hypercalcemia is transient and infrequent event with teriparatide or abaloparatide therapy and has been reported in 6.4% and 3.4% of patients, respectively (Miller et al. 2018). Consistently, we did not observe a significant difference in serum Ca^2+^ among animals treated with vehicle, abaloparatide, or teriparatide. The significant increase in serum Ca^2+^ detected when abaloparatide was compared to vehicle‐treated animals is consistent with the amplified PTHR1 signaling by abaloparatide treatment. The elevation in serum Pi induced by the higher doses of teriparatide and abaloparatide is probably related to the increased bone resorption and/or stimulation of Vitamin D‐mediated intestinal Pi resorption. It is important to emphasize that these findings reflect the effects of teriparatide and abaloparatide on mineral homeostasis in response to intermittent treatment and 24 h after the last injection. The classic hypercalcemic and hypophosphatemic actions of PTH have commonly and more accurately been assessed using multiple measurements during the first 0.5–24 h after drug injection (Maeda et al. 2013; Bi et al. 2016). This is, however, beyond the scope of this investigation.

Despite this major progress in clinical development of PTHR1‐based anabolic agents, there are still limitations and unmet needs, primarily the absence of other modes of administration causing lack of treatment adherence, and the restricted 2‐year anabolic window. To overcome these limitations and to design next generation of improved PTHR1 bone therapeutics, a better understanding of the cellular mechanisms that underlie the superior anabolic actions of abaloparatide over teriparatide should prove useful.

PTHR1 is a member of subclass B seven transmembrane spanning GTP‐binding protein (G‐protein) coupled receptors (GPCR) that activates multiple G‐proteins, primarily the Gs but also Gq/11, Gi, and G12/13 G‐proteins. As a result, PTHR1 activation stimulates multiple downstream pathways and events such as Gs/adenylate cyclase/cAMP/protein kinase A, Gq11/phospholipase C/diacylglycerol‐inositol‐triphosphate‐Ca/protein kinase C, phospholipase D, and Src/*β*‐arrestin/MAPK pathways and PTHR1 phosphorylation and internalization (Abou‐Samra et al. 1992; Sneddon et al. 2000; Tawfeek et al. 2001; Tawfeek et al. 2002; Ahmed et al. 2003; Sneddon et al. 2004; Tawfeek and Abou‐Samra 2004; Singh et al. 2005; Gesty‐Palmer et al. 2006; Rey et al. 2006; Sneddon and Friedman 2007; Sneddon et al. 2007; Tawfeek and Abou‐Samra 2008; Garrido et al. 2009; Tawfeek and Abou‐Samra 2012). Despite the complexity of PTHR1 signaling, only two pathways, namely Gs‐cAMP and *β*‐arrestin, have been implicated as the major mediators of PTH effects on bone remodeling. The role of Gs‐cAMP pathway was initially suggested using a mouse model of a constitutively active naturally occurring PTHR1 mutant (H223R). This mutant has been shown to enhance basal Gs/cAMP activity when expressed in cells and to subsequently cause great increase in trabecular bone turnover and volume whether expressed in osteoblasts or osteocytes (Calvi and Schipani 2000; Calvi et al. 2001; O'Brien et al. 2008; Rhee et al. 2011). Following studies further demonstrated that postnatal deletion of the G‐protein Gs alpha in the osteoblast lineage cells of male or female mice led to loss of daily PTH‐increased fractional trabecular bone volume and cortical bone thickness (Sinha et al. 2016). The role of *β*‐arrestin in PTHR1 osteoanabolic effects was suggested by investigations using a D‐Trp12, Tyr34 bovine PTH (7‐34), a signal selective PTH analog; this analog has been shown to stimulate PTHR1 recruitment of *β*‐arrestin/ERK1/2 signaling but to lack PTHR1 activation of G‐proteins signaling (Gesty‐Palmer et al. 2006). When injected daily to WT mice, D‐Trp12, Tyr34 bovine PTH (7‐34) produced an increase in bone formation and bone gain (Gesty‐Palmer et al. 2009; Bohinc and Gesty‐Palmer 2011). However, these effects were attenuated when the same analog was administered to *β*‐arrestin 2 knockout mice. Furthermore, Gq/11 pathway in osteoblast cells seems to act as a negative regulator of PTH stimulation of bone mass (Ogata et al. 2007; 2011). In this context, our findings demonstrate that abaloparatide activation of PTHR1 causes a reduction in EC50 values for both cAMP and *β*‐arrestin stimulation when compared to teriparatide. Accordingly, the greater anabolic effects of abaloparatide over teriparatide can be attributed, at least, in part to the abaloparatide‐improved PTHR1 signaling to both Gs/cAMP and *β*‐arrestin pathways. Recently, however, Ricarte et al. indicated that PTH (1–34) treatment produced higher stimulation of cAMP and its downstream PKA effectors than PTHrP (1‐36) or abaloparatide (Ricarte et al. 2018). Moreover, PTH was shown to induce larger RANKL gene expression in vivo and in vitro (Ricarte et al. 2018). The discrepancy between our findings and those of Ricarte et al is likely attributed to the different osteoblast cell systems (primary calvarial osteoblast vs. clonal MC3T3‐E1 cell line) and/or the methodology used in the two studies. On the other hand, the difference in RANKL results clearly reflects the complexity of the mechanisms regulating serum RANKL versus RANKL gene expression in osteoblast cells or in cells in bone extract. Alternatively, gene and its protein product levels do not necessarily correlate given that distinct regulatory mechanisms are involved in transcription and translation.

In conclusion, the present report demonstrates that the superior osteoanabolic effects of abaloparatide are likely attributed to the enhanced bone formation and the subsequent improvement of both cortical and trabecular bone thickness. The study further suggests that these favorable effects are mediated by the greater activation of both Gs‐cAMP and *β*‐arrestin signaling by abaloparatide.

## Conflict of Interest

The authors declare no competing financial interests.
